# The incidence, presentation, outcomes, risk of mortality and economic data of drug-induced liver injury from a national database in Thailand: a population-base study

**DOI:** 10.1186/s12876-016-0550-0

**Published:** 2016-10-28

**Authors:** Abhasnee Sobhonslidsuk, Kittiyod Poovorawan, Ngamphol Soonthornworasiri, Wirichada Pan-ngum, Kamthorn Phaosawasdi

**Affiliations:** 1 Division of Gastroenterology and Hepatology, Department of Medicine, Faculty of Medicine, Ramathibodi Hospital, Mahidol University, 270 Rama 6 road, Rajathevee, Bangkok 10400 Thailand; 2 Department of Clinical Tropical Medicine, Faculty of Tropical Medicine, Mahidol University, Rajathevee, Bangkok 10400 Thailand; 3 Department of Tropical Hygiene, Faculty of Tropical Medicine, Mahidol University, Rajathevee, Bangkok 10400 Thailand; 4 Vichaiyut Hospital and Medical Center, Rajathevee, Bangkok 10400 Thailand

**Keywords:** Drug-induced liver injury, Hepatitis, Cirrhosis, Acetaminophen, Anti-mycobacterial agents

## Abstract

**Background:**

Toxic liver diseases are mainly caused by drug-induced liver injury (DILI). We assessed incidences and outcomes of DILI including associated factors for mortality.

**Methods:**

We performed a population-based study of hospitalized patients with DILI. Information was retrieved from the Nationwide Hospital Admission Data using ICD-10 code of toxic liver diseases (*K71*) and additional codes (*T36*–*T65*). The associated factors were analyzed with log-rank test, univariate and multiple cox regression analysis.

**Results:**

During 2009–2013, a total of 159,061 (average 21,165 per year) admissions were related to liver diseases. 6,516 admissions (1,303 per year) were due to toxic liver diseases. The most common type of toxic liver disease was acute hepatitis (33.5 %). In-hospital and 90-day mortality rates were 3.4 % and 17.2 %. DILI with cirrhosis yielded the highest in-hospital and 90-day mortality rates (15.8 % and 47.4 %). Acetaminophen, cirrhosis and age ≥ 60 years were seen in 0.5 %, 8.3 % and 50.1 % of patients who died versus 5 %, 2.3 % and 32.4 % of survivors. Factors associated with mortality were cirrhosis (HR 2.72, 95 % CI: 2.33–3.19), age ≥60 years (HR 2.16, 95 % CI: 1.96–2.38), human immunodeficiency viral infection (HR 2.11, 95 % CI: 1.88–2.36), chronic kidney disease (HR 1.59, 95 % CI: 1.33–1.90), chronic obstructive pulmonary disease and bronchiectasis (HR 1.55, 95 % CI: 1.17–2.04), malnutrition (HR 1.43, 95 % CI: 1.10–1.86) and male (HR 1.31, 95 % CI: 1.21–1.43). Acetaminophen DILI yielded lower risks of mortality (HR 0.24, 95 % CI: 0.13–0.42). The most common causes of DILI were acetaminophen (35.0 %) and anti-tuberculous drugs (34.7 %).

**Conclusions:**

DILI is an uncommon indication for hospitalization carrying lower risks of death except in patients with non-acetaminophen, cirrhosis, elderly or concomitant diseases.

## Background

Liver injury which is caused by drugs and herbal supplement is an infrequent but increasing health problem. About 52 % of reported cases of acute liver failure (ALF) in the United States were related to drug-induced liver injury (DILI) [1]. The incidence of DILI in medical inpatients was estimated to be approximately 1 in 100 patients [2, 3]. However, the prevalence of DILI in Asia has not been well described [4]. From the Drug-Induced Liver Injury Network (DILIN) studies, 15.5 % of patients with liver injury were caused by herbal and dietary supplements [5]. In Asian countries, there have been conflicting reports of the number of herbal DILI [6, 7]. However, a subsequent systematic review in reported the incidence of DILI of 0.71 % after exposure to herbal remedies [7].

The International Classification of Disease (ICD) revision 10 system has been used in Thailand’s administrative and health care database for the identification of patient cohorts and financial works [8]. The ICD-10 code for toxic liver disease, or *K71* (ICD-10 codes are shown in italics all through this paper), is aimed to define drug-induced idiosyncratic (unpredictable) liver disease and drug-induced toxic (predictable) liver disease. Additional external cause codes (*T36*–*T65*) are often required after the coding of toxic liver diseases in order to identify the cause of DILI [9, 10]. Toxic liver diseases are mainly contributed by DILI [7]. Since 2002, the Universal Coverage Scheme has been implemented in Thailand for people whose health care cost is neither covered by the Social Health Insurance Scheme nor the Civil Servant Medical Benefit Scheme [11]. Currently, the Universal Coverage Scheme covers the health care cost of more than 47 million people (75 % of Thai population) [11]. To address the less well-described DILI in this region, we assessed incidences, presentation, outcomes and economic burden of hospitalized patients with DILI including the risk factors of mortality in Thai population from the large database of the Nationwide Hospital Admission Data.

## Methods

### Data source and study population

The study protocol was approved by the Committee on Human Rights related to Research Involving Human Subjects, Faculty of Medicine, Ramathibodi Hospital (ID 07–59–60) and it was carried out according to the Good Clinical Practice Guideline without obtaining inform consent. We performed a population-based study of hospitalized adult patients aged at least 19 years old with DILI whose health care cost was under the Universal Coverage Scheme in all 77 provinces to evaluate the incidences and outcomes of DILI including associated factors for mortality in Thailand. All data were retrospectively retrieved from the 2009 to 2013 Nationwide Hospital Admission Data from the National Health Security Office (NHSO), Thailand, which included more than 75 % of Thai population, by using the International Classification of Diseases, 10th edition (ICD-10) code indicative of toxic liver diseases (*K71*). Individual patients with one of the ICD-10 codes entered between January 1, 2009 and December 31, 2013 were identified. The diagnosis of DILI in the NHSO database was performed by physicians in their clinical practice without obligation to use specific and objective diagnostic criteria such as the Roussel Uclaf Causality Assessment Method (RUCAM) or its previous term, the Council for International Organization of Medical Sciences (CIOMS) [12], which can be the study limitation. However, the bias in the diagnosis of DILI was controlled by further examining additional external cause codes of ICD-10, chiefly the poisoning by drugs and biologic substances (*T36*–*T50*) and the toxic effects of substances from non-medicinal sources (*T51*–*T65*). In Thailand, hospitals are classified into three levels, i.e. primary, secondary and tertiary hospitals. The baseline characteristics, demographic data, length of hospital stay, admission cost, outcomes and causes of DILI were collected and analyzed.

### Availability of data and materials

All available raw data will not be shared as it consisted of confidential patient information that abide by the signed contract and regulation. All other relevant study data are presented in the tables.

### Statistical analysis

Continuous variables were compared among groups using one-way ANOVA and Kruskal Wallis tests as appropriate. Categorical variables were compared among groups using χ^2^ and Fisher’s exact test. Factors associated with mortality were analyzed with log-rank test, univariate and multiple cox regression analysis. The hazard ratio [HR] and 95 % confidence interval (CI) of each factor has been demonstrated. A P <0.05 was considered statistically significant. Statistical analysis was performed with SPSS version 13 (SPSS Inc., Chicago IL).

## Results

### Demographic and admission data of hospitalized patients with DILI

During 2009–2013, a mean of 5.6 million admissions from all causes occurred per year. A total of 159,061 admissions (or 21,165 admissions per year) were related to liver diseases. 6,516 admissions (or 1,303 admissions per year) were due to DILI (Table 1). The average annual admission rates of DILI were 4.1 % of all liver disease admissions and 0.12 % of the total admissions. The incidence rates of DILI did not significantly change over the study period (0.11 %, 0.11 %, 0.12 %, 0.12 %, 0.13 %, *P* = 0.058). The mean age of the study patients was 51.9 ± 18.6 years. The mean length of hospital stay was 6.7 ± 6.7 days. The average health care cost of admission of DILI was 533,955 ± 53,532 USD per year, which was about 5.4 % and 0.1 % of the health care cost of admission due to liver diseases (9,888,056 USD) and overall gastrointestinal diseases (391,512,096 USD).

**Table 1:** Annual incidence and demographic data of patients who were admitted with drug-induced liver injury (DILI) from 2009 to 2013 in Thailand

	2009	2010	2011	2012	2013	Average
Annual admissions, n	1,197	1,244	1,312	1,362	1,401	1,303
% of admissions (per liver diseases)	4.2	4.1	4.1	4.1	4.0	4.1
% of admissions (per total admissions)	0.11	0.11	0.12	0.12	0.13	0.12
Male, n (%)	603 (50)	634 (51)	677 (52)	720 (53)	724 (52)	671.6 (51.5)
Age, years	52.4 ± 18.5	52.2 ± 18.6	50.4 ± 18.5	52.7 ± 18.6	52.3 ± 18.8	52.0 ± 18.6
Length of hospital stay, days	6.9	6.8	6.9	6.5	6.4	6.7 ± 6.7
Cost of hospitalization, USD	443,230	535,600	545,650	565,661	579,636	533,955 ± 53,532
Mortality, n (%)						
‒ In-hospital mortality	34 (2.8)	44 (3.5)	52 (4.0)	47 (3.5)	42 (3.0)	43.8 (3.4)
‒ 90 day mortality	288 (24.0)	251 (20.0)	233 (18.0)	202 (15.0)	144 (10.0)	223.6 (17.2)

From the available recorded data (of 589 cases), the two most common causes of DILI were 4-aminophenol (*T39.1*) which is the primary degradation product of acetaminophen [13], and anti-mycobacterial drugs (*T37.1*, *T36.6*) (35 % and 34.6 %, respectively) (Table 2).

**Table 2:** The list of common drugs and substances as the causes of drug-induced liver injury (DILI) in 589 cases

Drug or substance	Number	%
Acetaminophen (*T39.1*)	206	35
Antimycobacterial drugs (*T37.1* and *T36.6*)	204	34.6
Other and unspecified drugs and substances (*T50.9*)	34	5.8
Antiviral drugs (*T37.5*)	22	3.7
Ingested mushroom (*T62.0*)	16	2.7
Herbicides and fungicides (*T60.3*)	13	2.2
Antihyperlipdemia and antiarteriosclerotic drugs (*T46.6*)	7	1.2
Aminoglycosides (*T36.5*)	6	1.0
Other antibiotics (*T36.8*)	6	1.0
Other nonsteroidal anti-inflammatory drugs (*T39.3*)	6	1.0
Local antifungal, anti-infective and anti-inflammatory drugs (*T49.0*)	6	1.0
Others	63	10.7

### The risks of in-hospital and 90 day mortality

The average in-hospital and 90-day mortality rates of DILI were 3.4 % and 17.2 %, which were lower than those of overall liver diseases (6.8 % and 29.2 %) (*P* < 0.001). The highest number of DILI was found in the northeastern region and primary health care centers (Tables 3 and 4). However, the greatest in-hospital mortality rates were seen in Bangkok and in patients who were admitted in tertiary health care centers despite the lowest number of case admission in these sectors.

**Table 3:** The number (%) of patients with drug-induced liver injury (DILI) and in-hospital mortality according to country regions

Country region	Number	%	In-hospital mortality (*n*)	In-hospital mortality rate (%)
Central	703	11.4	36	5.1
Northeast	2421	39.2	34	1.4
North	588	9.5	24	4.1
South	650	10.5	15	2.3
Other	1311	21.2	56	4.3
Bangkok	501	8.1	31	6.2
Total^a^	6174	100.0	196	3.2

^a^missing data in 2 patients

**Table 4:** The number (%) of patients with drug-induced liver injury (DILI) and in-hospital mortality according to hospital levels

Hospital level	Number	%	In-hospital mortality (n)	In-hospital mortality rate (%)
Primary level	2575	41.7	33	1.3
Secondary level	1960	31.7	84	4.3
Tertiary level	1639	26.5	79	4.8
Total^a^	6174	100.0	196	3.2

^a^missing data in 2 patients

### The distribution of DILI types

The three most common types of DILI were acute hepatitis (*K71.2*) (33.5 %), not elsewhere classified hepatitis (*K71.6*) (27.3 %) and hepatic necrosis (*K71.1*) (25.6 %) (Table 5). DILI that occurred on top of cirrhosis (*K71.7*) notably yielded the highest in-hospital and 90-day mortality rates (15.8 % and 47.4 %). DILI with chronic lobular hepatitis (*K71.4*) and cholestasis (*K71.0*) were the second and third patterns of DILI associated with the in-hospital mortality. Chronic active hepatitis (*K71.5*) and chronic lobular hepatitis (*K71.4*) were ranked the second and third types of DILI related to the 90-day mortality.

**Table 5:** The number (%) of patients and mortality rates classified according to the types of drug-induced liver injury (DILI)

Type of DILI	Number	%	In-hospital mortality (%)	90-day mortality (%)
Cholestasis (*K71.0*)	329	5.3	6.1	24.0
Hepatic necrosis (*K71.1*)	1580	25.6	4.2	21.7
Acute hepatitis (*K71.2*)	2072	33.5	2.2	17.3
Chronic persistent hepatitis (*K71.3*)	29	0.5	3.5	24.1
Chronic lobular hepatitis (*K71.4*)	13	0.2	7.7	30.8
Chronic active hepatitis (*K71.5*)	7	0.1	0.0	42.9
Hepatitis, not elsewhere classified (*K71.6*)	1685	27.3	2.7	19.5
Fibrosis and cirrhosis of liver (*K71.7*)	57	0.9	15.8	47.4
Other disorders of liver (*K71.8*)	46	0.7	2.2	30.4
Unspecified (*K71.9*)	358	5.8	1.4	18.2
Total	6176	100.0	3.2	19.9

### Independent factors associate with mortality in patients having DILI

**Fig. 1:**
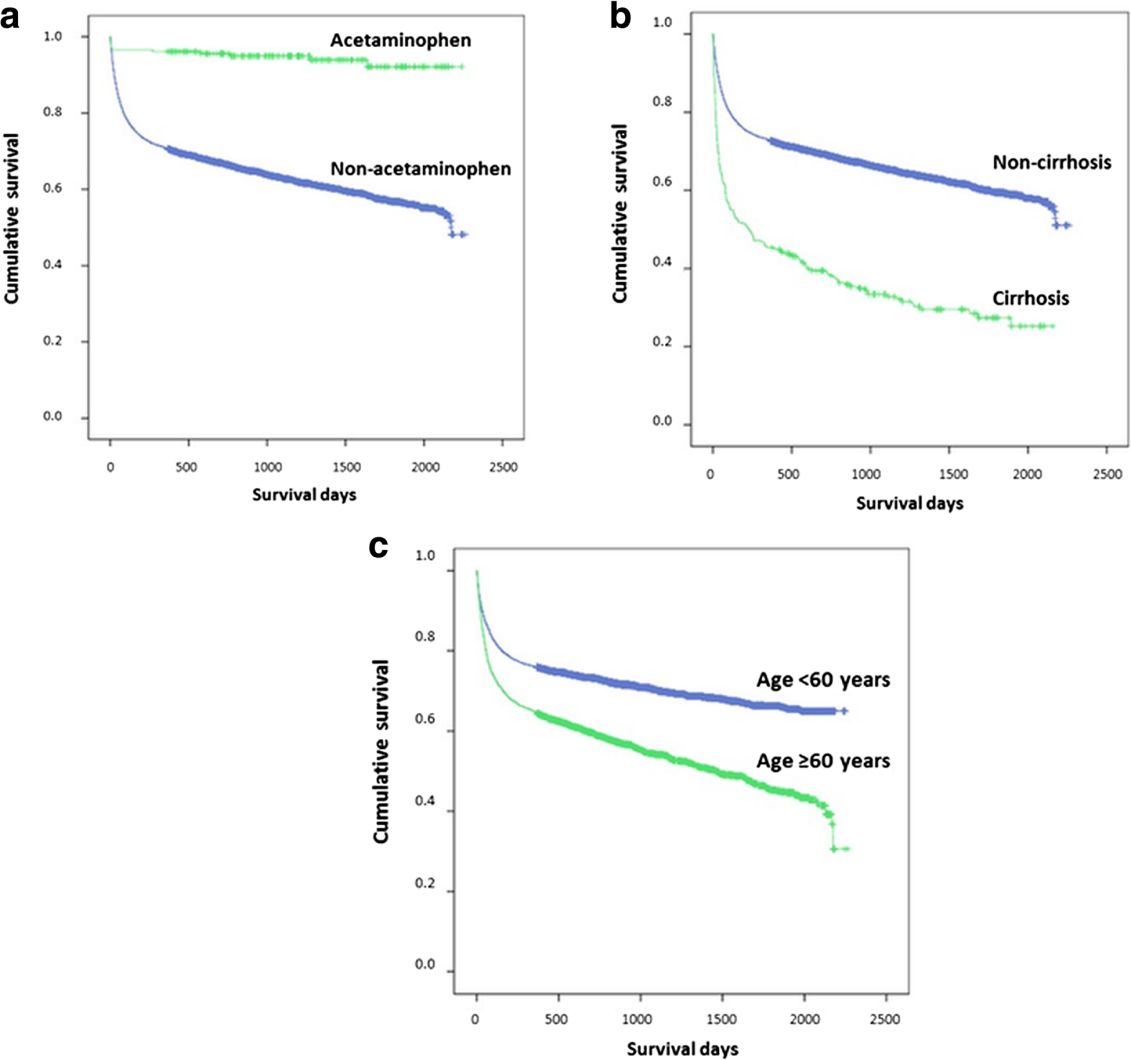
Cumulative survival of patients with drug-induced liver injury (DILI) in relation to acetaminophen or non-acetaminophen drugs as the cause of DILI, cirrhotic status and age. **a** Acetaminophen vs. non-acetaminophen. **b** Cirrhosis vs. non-cirrhosis. **c** Age ≥ 60 or < 60 years

Acetaminophen, cirrhosis and age ≥ 60 years were seen in 0.5 %, 8.3 % and 50.1 % of patients who died, comparing to 5 %, 2.3 % and 32.4 % of those who survived. Table 6 shows that the independent factors associate with mortality in DILI were acetaminophen (hazard ratio [HR] 0.24, 95 % confidence interval [CI]:0.13–0.42), cirrhosis (HR 2.72, 95 % CI: 2.33–3.19), age ≥60 years (HR 2.16, 95 % CI: 1.96–2.38), human immunedeficiency virus (HIV) (HR 2.11, 95 % CI: 1.88–2.36), chronic kidney disease (CKD) (HR 1.59, 95 % CI: 1.33–1.90), chronic obstructive pulmonary disease (COPD)/bronchiectasis (HR 1.55, 95 % CI: 1.17–2.04), malnutrition (HR 1.43, 95 % CI: 1.10–1.86), and being male (HR 1.31, 95 % CI: 1.21–1.43). The etiology of non-acetaminophen, cirrhosis and elderly (age ≥60) yielded significant impact on long-term survival (*P* < 0.001) (Fig. 1).

**Table 6:** Independent factors associate with mortality in drug-induced liver injury (DILI)

	Univariate Cox regression			Multiple Cox regression		
	Crude Hazard Ratio	95 % CI	p	Adjusted Hazard Ratio	95 % CI	*p*
Acetaminophen	0.13	0.07–0.22	<0.001	0.24	0.13–0.42	<0.001
Cirrhosis	2.60	2.24–3.02	<0.001	2.72	2.33–3.19	<0.001
Age ≥ 60 years	1.74	1.61–1.89	<0.001	2.16	1.96–2.38	<0.001
HIV	1.39	1.26–1.53	<0.001	2.11	1.88–2.36	<0.001
Chronic kidney diseases	1.94	1.63–2.32	<0.001	1.59	1.33–1.90	<0.001
COPD/bronchiectasis	1.92	1.46–2.53	<0.001	1.55	1.17–2.04	0.002
Malnutrition	1.87	1.44–2.42	<0.001	1.43	1.10–1.86	0.007
Male	1.41	1.29–1.53	<0.001	1.31	1.21–1.43	<0.001
Ascites	2.17	1.56–3.02	<0.001	1.40	0.97–1.98	0.052
Mushroom	0.14	0.00–1.03	0.053	0.22	0.03–1.53	0.125

*HIV* human immunodeficiency virus, *COPD* chronic obstructive pulmonary disease, *CI* confidence interval

## Discussion

From the Nationwide Hospital Admission Data of Thailand, about 1.2 of 100 admissions were due to DILI, which is close to the report from previous studies [2, 3]. The pattern of acute hepatitis or hepatocellular injury was the most common presentation of DILI in this study and others [2, 4, 14]. The outcome of cholestatic DILI was reportedly to be better than hepatocellular DILI.[2] Although, the cost of admission due to DILI was about 5.4 % of liver diseases and 0.1 % of overall gastrointestinal diseases, the in-hospital and 90-day mortality rates of DILI were about 3.4 % and 17.2 %. The highest in-hospital and 90-day mortality rates that were noticed in Bangkok and in tertiary care centers are explained by the policy of referral system of Thailand. From previous studies, jaundice, female sex, and hepatocellular injury were found to associate with the development of ALF, death and the need for liver transplantation [2, 15]. DILI patients who had liver cirrhosis, even without jaundice, had the highest risk of mortality in this study. The finding supports the significant impact of having pre-existing liver diseases on increasing severity of DILI and risks of mortality [16]. In addition, individual ages 60 or over and those with medical comorbidities were more likely to have higher mortality. These finding are in contrast to a conclusion from a previous study which showed that individuals 65 years or older did not have higher mortality as they were more likely to have cholestatic liver injury [16]. A lower risk of mortality in patients with acetaminophen-related DILI was revealed in this study. From a previous study, the mortality rate of acetaminophen-induced ALF was lower than non-acetaminophen-induced ALF (5.6 % vs. 21.4 %) [1]. A large retrospective cohort study in the United States demonstrated that acetaminophen, dietary/herbal supplements and antimicrobials were the common causes of drug-induced ALF in 56.3 %, 18.8 % and 6.3 %, respectively [1].

Many reports following prospective studies in the United States and Spain showed that antimicrobial agents especially amoxicillin-clavulanate were the most common drugs relating to DILI [2, 16, 17]. Based on the available data of 589 cases, the leading causes of DILI were acetaminophen and anti-tuberculous drugs. The common causes of DILI that were reported from Asian countries were anti-tuberculous drugs, antibiotics and anticonvulsants [4, 15]. Herbal remedies, or complementary and alternative medicines, have frequently been a common factor of DILI in this part of the world [4, 6, 14]. Herbal remedies were not found to be an important cause of DILI in Thai population due to a low number of herbal remedies recorded under the code *F55.1*. Nevertheless, we cannot conclude that herbal remedies do not contribute to DILI in Thailand.

As a retrospective study with nationwide database, our report has some limitation in term of data missing and the diagnostic criteria of DILI. The accuracy of data depends upon data input by health care workers. Using the ICD-10 code to identify DILI and adverse drug events may be subjected to substantial variability including false positive and false negative results [8, 9]. RUCAM, a well-established, structured and standardized tool to assess causality and diagnose in cases of suspected DILI and HILI [12], was not employed in this study. A further prospective study using RUCAM is needed to systematically investigate all aspects of DILI in Thailand.

## Conclusions

DILI is an uncommon clinical indication of hospitalization, but it carries higher risk of death in cirrhotic patients, elderly, male gender or having medical comorbidities. Our study provides a basis for future research in the field of DILI.

## Abbreviations

ALF: Acute liver failure
CI: Confidence interval
CKD: Chronic kidney disease
COPD: Chronic obstructive pulmonary diseases
DILI: Drug-induced liver injury
HIV: Human immunedeficiency virus
HR: Hazard ratio
ICD: International classification of disease
NHSO: National health security office

## References

[1] Goldberg DS, Forde KA, Carbonari DM, Lewis JD, Leidl KB, Reddy KR (2015). Population-representative incidence of drug-induced acute liver failure based on an analysis of an integrated health care system. Gastroenterology.

[2] Andrade RJ, Lucena MI, Fernandez MC, Pelaez G, Pachkoria K, Garcia-Ruiz E (2005). Drug-induced liver injury: an analysis of 461 incidences submitted to the Spanish registry over a 10-year period. Gastroenterology.

[3] Meier Y, Cavallaro M, Roos M, Pauli-Magnus C, Folkers G, Meier PJ (2005). Incidence of drug-induced liver injury in medical inpatients. Eur J Clin Pharmacol.

[4] Zhou Y, Yang L, Liao Z, He X, Zhou Y, Guo H (2013). Epidemiology of drug-induced liver injury in China: a systematic analysis of the Chinese literature including 21,789 patients. Eur J Gastroenterol Hepatol.

[5] Navarro VJ, Barnhart H, Bonkovsky HL, Davern T, Fontana RJ, Grant L (2014). Liver injury from herbals and dietary supplements in the U.S. Drug-Induced Liver Injury Network. Hepatology.

[6] Suk KT, Kim DJ, Kim CH, Park SH, Yoon JH, Kim YS (2012). A prospective nationwide study of drug-induced liver injury in Korea. Am J Gastroenterol.

[7] Oh SJ, Cho JH, Son CG (2015). Systematic review of the incidence of herbal drug-induced liver injury in Korea. J Ethnopharmacol.

[8] Hohl CM, Karpov A, Reddekopp L, Doyle-Waters M, Stausberg J (2014). ICD-10 codes used to identify adverse drug events in administrative data: a systematic review. J Am Med Inform Assoc.

[9] Stausberg J, Hasford J (2010). Identification of adverse drug events: the use of ICD-10 coded diagnoses in routine hospital data. Dtsch Arztebl Int.

[10] Myers RP, Leung Y, Shaheen AA, Li B (2007). Validation of ICD-9-CM/ICD-10 coding algorithms for the identification of patients with acetaminophen overdose and hepatotoxicity using administrative data. BMC Health Serv Res.

[11] Limwattananon S, Tangcharoensathien V, Tisayaticom K, Boonyapaisarncharoen T, Prakongsai P (2012). Why has the Universal Coverage Scheme in Thailand achieved a pro-poor public subsidy for health care?. BMC Public Health.

[12] Danan G, Teschke R. RUCAM in Drug and Herb Induced Liver Injury: The Update. Int J Mol Sci. 2016;17. 10.3390/ijms17010014 PMC4730261 26712744

[13] Bloomfield MS (2002). A sensitive and rapid assay for 4-aminophenol in paracetamol drug and tablet formulation, by flow injection analysis with spectrophotometric detection. Talanta.

[14] Wai CT, Tan BH, Chan CL, Sutedja DS, Lee YM, Khor C (2007). Drug-induced liver injury at an Asian center: a prospective study. Liver Int.

[15] Devarbhavi H, Dierkhising R, Kremers WK, Sandeep MS, Karanth D, Adarsh CK (2010). Single-center experience with drug-induced liver injury from India: causes, outcome, prognosis, and predictors of mortality. Am J Gastroenterol.

[16] Chalasani N, Bonkovsky HL, Fontana R, Lee W, Stolz A, Talwalkar J (2015). Features and Outcomes of 899 Patients With Drug-Induced Liver Injury: The DILIN Prospective Study. Gastroenterology.

[17] Chalasani N, Fontana RJ, Bonkovsky HL, Watkins PB, Davern T, Serrano J (2008). Causes, clinical features, and outcomes from a prospective study of drug-induced liver injury in the United States. Gastroenterology.

